# Bilateral Endogenous Klebsiella pneumoniae Endophthalmitis in Culture-Negative Liver Abscess Requiring Evisceration: A Case Report and Review of Literature

**DOI:** 10.7759/cureus.36965

**Published:** 2023-03-31

**Authors:** Mohamed Noor Arjamilah, Mohd Yazid Aiman-Mardhiyyah, Ismail Shatriah, Evelyn Tai Li Min, Qi Zhe Ngoo

**Affiliations:** 1 Department of Ophthalmology and Visual Science, School of Medical Sciences, Health Campus, Universiti Sains Malaysia, Kubang Kerian, MYS; 2 Ophthalmology Clinic, Hospital Universiti Sains Malaysia, Kubang Kerian, MYS

**Keywords:** evisceration, non-diabetic, liver abscess, klebsiella pneumoniae, endogenous endophthalmitis

## Abstract

Endogenous endophthalmitis is a very rare but potentially devastating intraocular inflammation resulting from hematogenous dissemination into the eye from a remote focus of infection. We present a case of a 49-year-old Vietnamese gentleman with underlying hypertension and ischemic heart disease who presented with sudden onset bilateral eye blurring of vision for five days associated with fever, chills, and rigors. He started to have a chesty cough with right-sided pleuritic chest pain for three days as well as shortness of breath, which developed one day prior to admission. Bilateral ocular examinations and B-scan ultrasonography were consistent with endophthalmitis. A systemic workup was performed and showed multiloculated liver abscess and right lung empyema seen radiologically. Bilateral eye vitreous tap and intravitreal antibiotic injection were performed. He underwent ultrasound-guided pigtail catheter insertion and drainage of the subcapsular and pelvic collection. Microbiological findings revealed *Klebsiella pneumoniae* infection obtained from vitreous and endotracheal aspirate samples. There were no cultures yielded from the intraabdominal collection and peripheral blood. The right eye infection rapidly progressed to panophthalmitis, which subsequently led to globe perforation despite prompt treatment and eventually required evisceration. Thus, despite culture-negative pyogenic liver abscess in a non-diabetic patient, a high index of suspicion, emergent radiographic evaluation, and prompt intervention and treatment are crucial in salvaging the globes.

## Introduction

Endogenous endophthalmitis (EE) results from the metastatic spread of an organism from a primary site of infection. It is postulated that septic embolus enters the posterior vasculature and disseminates the organism into surrounding tissues after crossing the blood-ocular barrier. It is estimated that EE accounts for 2-15% of all cases of endophthalmitis [1], with hepatobiliary tract infection being the most common site of infection (90%), followed by urinary tract and lung infection [2].

In recent years, many cases have been reported in Asia. It can be due to a variety of causative organisms, which varies depending on the geographical location [3]. In Malaysia, Gram-negative organisms accounted for 50.6% of which* Klebsiella pneumoniae* was the most common organism isolated and accounted for 32.5% [4]. *Klebsiella* species are facultative anaerobic Gram-negative bacilli, which can be found in the gastrointestinal and nasopharyngeal as normal flora. This organism has the potential to evolve into a highly virulent bacteria, resulting in devastating endophthalmitis. This ability is further enhanced among patients with poorly controlled diabetes mellitus [5]. The variable final visual outcome is commonly seen among patients following EE caused by *Klebsiella pneumoniae* and is often disappointing.

Here, we report a case of culture-negative pyogenic liver abscess in a non-diabetic patient who presented with bilateral endogenous *Klebsiella pneumoniae* endophthalmitis which required evisceration in one eye despite prompt treatment.

## Case presentation

A 49-year-old Vietnamese male with underlying hypertension and ischemic heart disease presented with sudden onset bilateral eye blurring of vision for a duration of five days associated with fever, chills, and rigors. He started to experience chesty cough and right-sided pleuritic chest pain for three days, as well as shortness of breath, which developed one day prior to admission.

**Figure 1:**
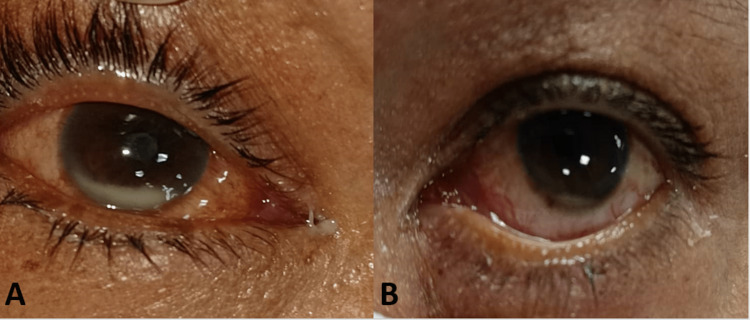
A) Right anterior segment at presentation showing conjunctival congestion, mild chemosis nasally, hypopyon approximately one-third of the anterior chamber, and fibrinous membrane covering the pupil. B) Left eye anterior segment at presentation showing mild conjunctival congestion with streak of hypopyon.

The visual acuity was perception to light at all four quadrants in the right eye and hand movement in the left eye. The relative afferent pupillary defect was positive in the right eye. The ocular motility was full in all directions of both eyes. There was no proptosis or eyelid swelling in both eyes. Anterior segment examination of the right eye showed marked conjunctival congestion with mild chemosis nasally. There was hypopyon at approximately one-third of the anterior chamber, a fibrinous membrane covering the pupil, and 360-degree posterior synechiae (Figure 1A). There was no view of the fundus.

Examination of the left eye showed generalized conjunctival congestion with no chemosis. There was a streak of hypopyon seen, fibrinous membrane at the pupillary margin sparing at the 12 o’clock position but there were no posterior synechiae (Figure 1B). The posterior segment of the left eye showed dense vitritis. Intraocular pressure of the right and left eyes was 9 mmHg and 6 mmHg, respectively. B-scan ultrasonography (US) of the right eye showed retinal detachment with dense vitritis and loculation while the left eye showed a flat retina with dense vitritis and the presence of loculations.

**Figure 2:**
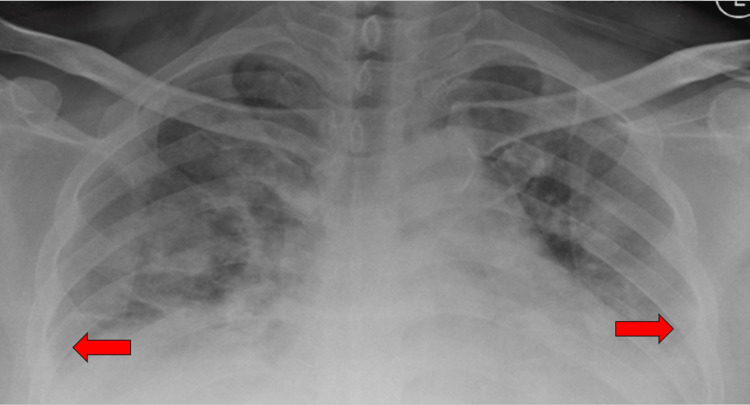
Chest x-ray showing bilateral blunted costophrenic angles with pneumonic changes.

Systemic examinations revealed the presence of coarse crepitation up to the mid zone in both lungs. There was no abdomen tenderness, distension, or organomegaly. He was initially diagnosed with community-acquired pneumonia with parapneumonic effusion based on initial clinical findings supported by a chest x-ray that was suggestive of pneumonia with blunted both costophrenic angles (Figure 2). However, on the fourth day of his hospitalization, he developed sudden onset of abdominal pain associated with abdominal distension. The following day, he developed respiratory distress, necessitating intubation. He was intubated for 12 days and was placed in the intensive care unit.

**Figure 3:**
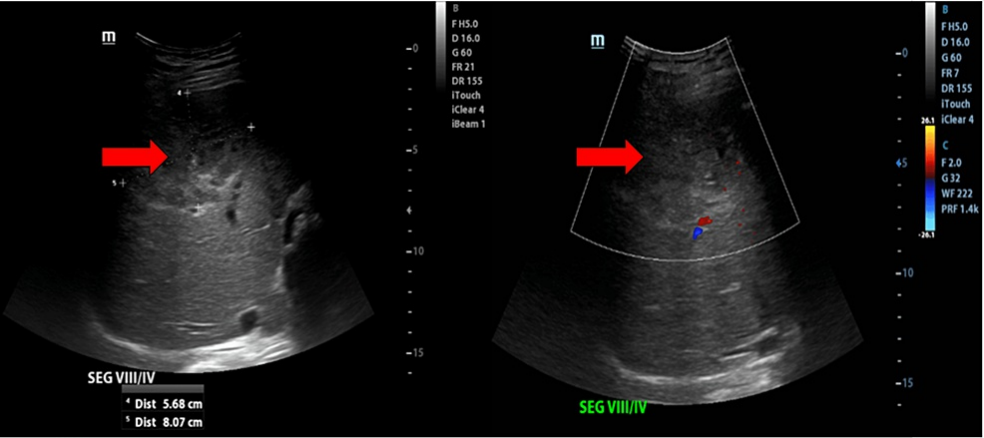
Ultrasound abdomen showing non-liquefied liver abscess at segment IV/VIII measuring 5.7cm x 8.1cm (AP x W).

**Figure 4:**
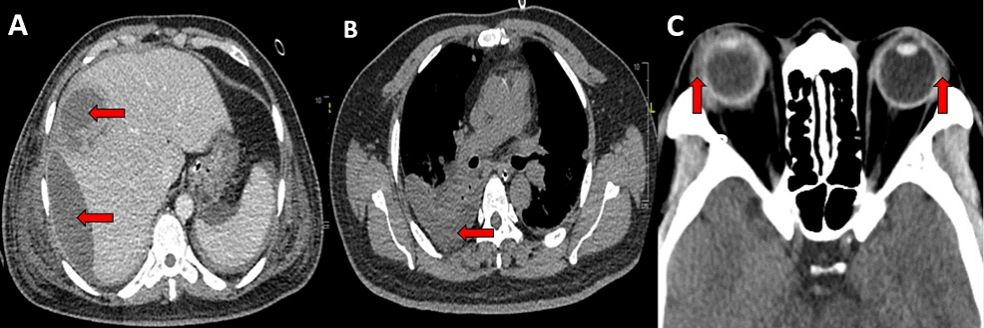
Contrast-enhanced computed tomography (CECT) scans A) CECT liver 4-phase shows irregular multiloculated hypodense collection at segment V/VIII suggestive of ruptured multiloculated liver abscess. B) CECT thorax shows loculated right pleural effusion with enhancing pleural outline representing empyema. C) CECT orbits shows irregular and enhancing scleral thickening in both globes with periorbital soft tissue thickening suggestive of panophthalmitis.

On radiological evaluation, urgent US abdomen was performed on day six of admission and showed the presence of a liver lesion at segment IV/VIII suggestive of non-liquefied liver abscess (Figure 3). The computed tomography (CT) liver 4-phase and contrasted CT thorax, abdomen, and pelvis was performed on day 13 of admission in view of worsening abdominal distension. The CT findings showed ruptured multiloculated liver abscess with multiloculated intrabdominal collection and multiple lung lesions suggestive of empyema (Figure 4A and Figure 4B). CT scan of brain and orbits showed bilateral panophthalmitis and optic neuritis as evidenced by the presence of generalized scleral thickening, vitreous humor hyperdensity, extensive fat streakiness involving the intraorbital space, and bilateral optic nerves thickening (Figure 4C).

On laboratory evaluation, full blood count showed elevated white blood cells count (15.69 x 10^9/L) with predominantly leucocytes (89.1%). The C-reactive protein (CRP) and erythrocyte sedimentary rate (ESR) were also elevated (>200mg/L and 78mm/60min respectively). Hemoglobin A1c (HbA1c) was normal (5.2%). There was no renal impairment and deranged liver enzymes. Microbiological findings revealed* Klebsiella pneumoniae* infection obtained from vitreous and endotracheal aspirate samples, which was sensitive to amoxycillin-clavulanate and cefuroxime, but resistant to ampicillin. There were no cultures yielded from the intraabdominal collection, peripheral blood, and urine samples.

The patient underwent ultrasound-guided pigtail catheter insertion and drainage of the subcapsular and pelvic collections and drained out 120cc pus and 140cc straw-colored fluid respectively. However, there was no culture yielded from both collections. Empyema of the right lung was evident radiologically; however, it was not feasible for drainage in view of multiple loculated lesions.

He underwent right eye evisceration due to worsening ocular condition leading to panopthalmitis and eventually spontaneous right globe perforation despite multiple intravitreal injections (vancomycin 2 mg/0.1 ml and ceftazidime 2 mg/0.1 ml) performed in both eyes. There was a scleral perforation at the 12 o’clock position 3 mm from limbus as well as corneal perforation from the 6 o’clock to 12 o’clock position with copious amount of pus discharge noted intraoperatively.

The patient completed intravenous (IV) meropenem 1 gram every eight hours for one week, IV meropenem 2 grams every eight hours for two weeks, IV UNASYN® (ampicillin and sulbactam) 9 grams every eight hours for two weeks, IV metronidazole 500 milligrams every eight hours for three weeks, IV Tazocin (piperacillin and tozobactam) 4.5 grams every six hours for two weeks and was discharged with oral Augmentin (amoxicillin and clavulanic acid) 625 milligrams every eight hours for four weeks.

At two months follow-up, the right eye wound was well healed. The visual acuity in the left eye remained poor with no perception of light at all four quadrants.

## Discussion

Endogenous *Klebsiella pneumoniae* endophthalmitis (EKE) is a devastating metastatic ocular infection, particularly in immunocompromised patients, with the liver being the most common site of infection (19%) [6]. Only a few cases in immunocompetent patients have been reported. Table 1 summarizes nine published cases of EKE from 2019 to 2021 and our case.

**Table 1:** Summary of published case reports of endogenous Klebsiella pneumoniae endophthalmitis OD, oculus dexter (right eye); OS, oculus sinister (left eye); OU, oculus uterque (both eyes); CF, counting finger; HM, hand motion; PL, perception to light; NPL, no perception to light; N/A, not available; (+), positive culture result; (-), negative culture result

Case reports	Age	Gender	Comorbidity	Liver abscess	Culture Result			Treatment	Laterality	Initial visual acuity	Final visual outcome
					Vitreous	Blood	Liver Abscess				
Al-Amri et al., 2010 [7]	55	Male	Diabetes mellitus	Present	+ - N/A			Intravitreal antibiotics injection, intravenous antibiotic, pars plana vitrectomy	OS	NPL	NPL
Al-Mahmood et al., 2011 [8]	43	Male	Diabetes mellitus	Present	+ - +			Intravitreal antibiotics injection, intravenous antibiotic	OS	PL	NPL
	70	Female	Diabetes mellitus, ischemic heart disease, hypertension, bronchial asthma	Present	+ - N/A			Intravitreal antibiotics injection, Intravenous antibiotics, left eye pars plana lensectomy-vitrectomy, right eye evisceration	OU	PL	OD: NPL OS: PL
Fujita et al., 2019 [9]	70	Female	Nil	Present	+ + +			Phacoemulsifications and aspiration and vitrectomy, intravenous antibiotics, ophthalmectomy	OD	PL	N/A
	50	Male	Diabetes mellitus, chronic thyroiditis	Present	+ N/A N/A			Phacoemulsifications and aspiration and vitrectomy, intravenous antibiotics	OD	PL	PL
Lim et al., 2020 [10]	50	Female	Diabetes mellitus	Present	N/A + +			Intravitreal antibiotics injection, intravenous antibiotics, pars plana vitrectomy	OS	HM	6/75
	62	Female	Diabetes mellitus	Present	- - -			Intravitreal antibiotics injection, intravenous antibiotics, pars plana vitrectomy	OD	CF	6/12
Zhao et al., 2021 [11]	80	Male	Diabetes mellitus, hypertension, coronary heart disease	Present	+ + +			Intravitreal antibiotics injection, right eye evisceration	OU	PL	OD: N/A OS: NPL
Correia et al., 2021 [12]	Middle-aged	Female	Nil	Present	- - -			Intravitreal antibiotics injection, intravenous antibiotics	OD	PL	PL
Current case (2023)	49	Male	Hypertension, ischemic heart disease	Present	+ - -			Intravitreal antibiotics injection, intravenous antibiotics, right eye evisceration	OU	OD: PL OS: HM	OS: NPL

The presenting age ranged between 43 and 80 years, with five cases being 50 years of age or younger and four cases being older than 60 years of age. Both genders were equally affected in previously reported cases. Seven out of nine published cases had underlying diabetes mellitus, while only two cases were non-diabetic and had no other comorbidities [7-12]. Our patient and a patient reported by Correia et al. were in the middle-aged group and had no underlying diabetes mellitus [12].

Diabetes mellitus remains one of the major risk factors for EE. It carries a poor prognosis in most patients, especially where Gram-negative organisms are involved [13]. In contrast, patients in our case and a case reported by Correia et al. had no underlying diabetes mellitus but still had subpar final visual outcomes. We believed that the delay to identify the source of infection is one of the contributing factors. In the case reported by Correia et al., their patients were initially assumed to have sepsis with no determined origin and uveitis of the right eye prior to the correct diagnosis of EKE being made. Abdominal US and abdominal CT were performed and a diagnosis of hydatid cyst was presumed initially until another re-evaluation with abdominal US and abdominal CT were performed and showed the presence of liver abscess. Even though their patients were treated with IV antibiotics before the correct diagnosis of EKE, the treatments were considered incomplete eventually leading to rapid deterioration of visual acuity [12]. In our case, the patient started to develop abdominal pain and distension on day six after being admitted, and an urgent abdominal ultrasound revealed our patient had a liver abscess. Therefore, the lesson to be learned here is that we should alert the physicians that an abdominal US should be performed as soon as possible in patients who have signs of sepsis and clinical evidence of endophthalmitis to look for the source of infection. 

The primary source of infection in all reported cases as shown in Table 1 was liver abscess. Culture and sensitivity tests were performed on blood, vitreous, and liver abscess samples in all cases. In two cases, all cultures were positive for *Klebsiella pneumoniae* (22%) [9,11], while in the other two cases, all cultures were negative [10,12]. For the vitreous samples, 67% were positive for *Klebsiella pneumoniae,* 22% were negative, and 11% of the results were unavailable [7-12]. For the blood samples, only 33% were positive, while 56% of the results were negative and 11% of the results were negative for *Klebsiella pneumoniae* [7-12]. For abscess fluid samples, 45% were positive for *Klebsiella pneumoniae*, 22% were negative, and 33% of the results were unavailable [7-12].

In our case, *Klebsiella pneumoniae* was isolated from vitreous samples only, while cultures from peripheral blood and abscess samples were negative. This is consistent with Ratra et al. who reported that ocular fluid samples tended to give positive culture results more than blood (58.6% vs 3.4%) as all patients with suspected EE immediately underwent an aqueous or vitreous tap before any intravitreal therapy [14]. Previously reported cases showed that only 45% of the cases had positive culture results from abscess fluid, which is consistent with Lo et al. who reported that only 60.1% of 741 patients with a pyogenic liver abscess had positive pus cultures [15]. This is likely because IV antibiotics are almost always prescribed prior to drainage, as in our case. This suggests that, despite culture-negative results, clinical evaluation is critical in establishing the diagnosis and initiating early treatment.

Previously reported cases as shown in Table 1 were treated with a combination of systemic and intravitreal antibiotics in which third-generation cephalosporins, second-generation cephalosporins, aminoglycosides, antifungals, and nitroimidazole were the antibiotics of choice administered [7-12]. Pars plana vitrectomy (PPV) was performed in four cases [7-10]. There were three unfortunate cases were required evisceration despite prompt treatment administered [8-11]. Intravitreal antibiotics are generally essential in treating bacterial EE as most topical or systemic antibiotics do not reach a sufficient therapeutic level in the vitreous [16]. PPV is another important treatment option; Zhang et al. have demonstrated that PPV can result in an 85% anatomical success rate with 80% retaining a vision of counting finger or better after surgery [17]. Furthermore, PPV reduces the likelihood of requiring enucleation or evisceration as it helps to reduce the microorganism load in the posterior segment, reduces the incidence of retinal detachment, and has a better glove salvage rate. However, early PPV was not feasible in our case due to the patient's systemic instability, as at that particular time, our patient was suffering from sepsis and required ventilatory support, posing a risk to his life. This could have been a factor in the final necessity of evisceration.

The prognosis of EKE in the course of a liver abscess is often disappointing. According to previous literature, 44-69% of eyes with EKE have a final visual acuity less than counting fingers, and 16-40% of eyes necessitate evisceration or enucleation [6,18]. Shelat et al. reported patients with culture-negative pyogenic liver abscess as having the same outcomes as those with *Klebsiella pneumoniae *pyogenic liver abscess [19].

In our review of previous literature, only 22% had final visual acuity of 6/12 or better, while 78% had perception to light or worse [7-12]. Lim et al. reported a favorable visual outcome as a result of early initiation of PPV for their patients with presenting visual acuity of hand motion or better [10]. The poor final visual acuity in spite of prompt and appropriate management could be because of the virulency of the organisms with possible high antibiotic resistance patterns as well as poor visual acuity at initial presentation, as in our case.

## Conclusions

A strong index of suspicion and urgent radiological evaluation is crucial in the diagnosis of EKE associated with liver abscess. The overall outcomes of patients with culture-negative pyogenic liver abscess are comparable to those of patients with* Klebsiella pneumoniae* pyogenic liver abscess even though they have no underlying diabetes mellitus, and this subsequently can lead to disastrous complications like globe perforation and necessitated evisceration.
